# Growth of malignant extracranial tumors alters microRNAome in the prefrontal cortex of TumorGraft mice

**DOI:** 10.18632/oncotarget.19835

**Published:** 2017-08-03

**Authors:** Anna Kovalchuk, Yaroslav Ilnytskyy, Rocio Rodriguez-Juarez, Amanda Katz, David Sidransky, Bryan Kolb, Olga Kovalchuk

**Affiliations:** ^1^ Department of Neuroscience, University of Lethbridge, Lethbridge, AB, Canada; ^2^ Department of Biological Sciences, University of Lethbridge, Lethbridge, AB, Canada; ^3^ Department of Oncology, Champions Oncology, Baltimore, MD, USA; ^4^ Leaders in Medicine Program, Cumming School of Medicine, University of Calgary, Calgary, AB, Canada

**Keywords:** chemo brain, tumor brain, epigenetics, microRNA, Gerotarget

## Abstract

A wide array of central nervous system complications, neurological deficits, and cognitive impairments occur and persist as a result of systemic cancer and cancer treatments. This condition is known as chemo brain and it affects over half of cancer survivors. Recent studies reported that cognitive impairments manifest before chemotherapy and are much broader than chemo brain alone, thereby adding in tumor brain as a component. The molecular mechanisms of chemo brain are under-investigated, and the mechanisms of tumor brain have not been analyzed at all. The frequency and timing, as well as the long-term persistence, of chemo brain and tumor brain suggest they may be epigenetic in nature. MicroRNAs, small, single-stranded non-coding RNAs, constitute an important part of the cellular epigenome and are potent regulators of gene expression. miRNAs are crucial for brain development and function, and are affected by a variety of different stresses, diseases and conditions. However, nothing is known about the effects of extracranial tumor growth or chemotherapy agents on the brain microRNAome.

We used the well-established TumorGraft ™ mouse models of triple negative (TNBC) and progesterone receptor positive (PR+BC) breast cancer, and profiled global microRNAome changes in tumor-bearing mice upon chemotherapy, as compared to untreated tumor-bearing mice and intact mice. Our analysis focused on the prefrontal cortex (PFC), based on its roles in memory, learning, and executive functions, and on published data showing the PFC is a target in chemo brain.

This is the first study showing that tumor presence alone significantly impacted the small RNAome of PFC tissues. Both tumor growth and chemotherapy treatment affected the small RNAome and altered levels of miRNAs, piRNAs, tRNAs, tRNA fragments and other molecules involved in post-transcriptional regulation of gene expression. Amongst those, miRNA changes were the most pronounced, involving several miRNA families, such as the miR-200 family and miR-183/96/182 cluster; both were deregulated in tumor-bearing and chemotherapy-treated animals. We saw that miRNA deregulation was associated with altered levels of brain-derived neurotrophic factor (BDNF), which plays an important role in cognition and memory and is one of the known miRNA targets. BDNF downregulation has been associated with an array of neurological conditions and could be one of the mechanisms underlying tumor brain and chemo brain. In the future our study could serve as a roadmap for further analysis of cancer and chemotherapy’s neural side effects, and differentially expressed miRNAs should be explored as potential tumor brain and chemo brain biomarkers.

## INTRODUCTION

Initial reports on the cognitive changes associated with cancer chemotherapies appeared in the late 1970s to the mid-1980s, but received scientific attention starting only in the mid-1990s. Several studies reported significant cognitive changes in cancer survivors [1]. These changes included problems with concentration, learning and memory, and executive functions. Central nervous system (CNS) toxicity manifestations had major negative effects on patient’s quality of life. Most of the chemotherapy-induced cognitive deficits were reported in breast cancer patients, in which deficits affected up to 50% of survivors and lasted for more than 10 years [2]. These effects were so significant that breast cancer survivors even coined a term for them—“chemo fog” or “chemo brain” [3, 4]. The latter term is now widely accepted and used to describe the CNS toxicity of chemotherapy (reviewed in [3-5]). A large-scale study following 200 breast cancer survivors for 21 years post chemotherapy reported on the long-term persistence of changes in executive functioning, verbal memory, and processing speed; likewise, they saw reductions of grey matter volume, and changes in white matter microstructural integrity [6-8]. While the majority of chemo brain reports come from the analysis of breast cancer survivor cohorts, chemo brain has also been reported in lymphoma, leukemia, lung, gastrointestinal, and other cancers, and has been accepted as a form of general post-chemotherapy CNS toxicity [4, 9].

The clinical analysis of chemo brain’s molecular mechanisms is complicated due to varying treatment protocols, significant inter-patient variability (as a result of co-morbidities), and various other factors [10-12]. Hence, the majority of chemo brain mechanistic studies used cell lines and rodent models (reviewed in [13, 14]), to determine that chemotherapy caused oxidative stress, suppressed neuronal proliferation and differentiation, induced apoptosis, affected the levels of histone modification and chromatin remodeling, caused the aberrant expression of genes, and altered the levels of neurotrophic and neurogenic proteins [15-17]. These molecular changes were associated with altered neurogenesis and deficits in learning and memory processes [16, 18-20].

Recent studies reported that cognitive impairments manifest before chemotherapy in 20%-30% of breast cancer patients and that these phenomena might be much broader than chemo brain alone. They may instead constitute both cancer and cancer treatment-associated cognitive changes reviewed in [5], thereby adding in the tumor brain component. While these studies provided significant insights into chemo brain, much remains to be learned about its molecular and cellular mechanisms. The molecular mechanism of tumor brain has not been analyzed at all.

The frequency and timing, as well as the long-term persistence, of chemo brain and tumor brain suggest they are epigenetic in nature. Epigenetic changes encompass meiotically heritable and mitotically stable alterations that regulate gene expression and genome stability; these include DNA methylation, histone modification, and non-coding RNA regulation [21]. Among small non-coding RNAs, microRNAs (miRNAs) are the most studied. MiRNA loci are transcribed by polymerase II, which gives rise to primary miRNA transcripts (pri-miRNAs). Portions of pri-miRNAs form hairpin structures that are cleaved and released by the action of ribonuclease Drosha, producing precursor miRNAs (pre-miRNAs). Next, pre-miRNAs are exported to the cytoplasm with the help of the Exportin 5 system, where the ribonuclease Dicer cleaves them, yielding mature single-stranded miRNA. Together with RNA-induced silencing complex (RISC) proteins such as Ago2, miRNAs interact with their associated mRNAs, thereby regulating the production of proteins. MiRNAs are crucial for brain development, neuronal differentiation, and axon regeneration following injury [22-25]. They regulate neuronal plasticity, and are likewise involved in the regulation of learning and memory [26-28]. Interestingly, miRNA deregulation was reported in autism [29], Alzheimer’s and Parkinson’s diseases, traumatic brain injury, stroke, amyotrophic lateral sclerosis, schizophrenia, and many other diseases and conditions [30-35]. Dicer and RISC proteins are important for brain development [36-38], and alterations in the expression of miRNA-processing machinery and RISC members are associated with brain diseases and conditions [39-42]. Furthermore, miRNA-based therapies may provide novel approaches for the treatment of neurodegenerative diseases [43], and miRNA profiles constitute essential diagnostic and prognostic biomarkers of diseases and conditions [44-46]. Various stresses and exposures affect brain microRNAome [47-49]. However, nothing is known about the effects of extracranial tumor growth or chemotherapy agents on the brain microRNAome.

Analyzing these phenomena in tumor-bearing animals is needed to gain a full mechanistic understanding of both chemo brain and tumor brain. In a previous study, we analyzed gene expression and DNA methylation changes in the PFC tissues of triple negative breast cancer (TNBC) and progesterone positive breast cancer (PR+BC) mice. We noted that tumor growth caused changes in gene expression Aging, in press (2017). We used mouse TumorGraft ™ models of untreated TNBC and PR+BC, as well as TNBC TumorGraft animals treated with Doxorubicin/ Cyclophosphamide/ Paclitaxel (TNBC/DCP) and PR+BC animals treated with Topotecan (PR+BC/TOP) or Crizotinib (PR+BC/CRIZ) to analyze the roles of miRNAs in tumor brain and chemo brain.

Here we report that extracranial malignant tumor growth had a profound effect on the microRNAome of the prefrontal cortex of experimental animals and caused changes in the levels of the miRNA processing machinery protein Ago2. Chemo and tumor brain-induced miRNA changes involved several miRNA families, such as the miR-200 family and miR-183/96/182 cluster, which were deregulated in PR+BC tumor-bearing and chemotherapy-treated animals. MiRNA deregulation was associated with altered levels of BDNF (brain-derived neurotrophic factor), a miRNA target that plays a key role in cognition and memory. Furthermore, deregulated miRNAs may serve as biomarkers of tumor brain and chemo brain.

## RESULTS

### Descriptive statistics of the next generation sequencing (NGS)

The NGS approach offers excellent technological opportunities to capture the entire repertoire of small RNAs and conduct a comprehensive analysis of the small RNAome [50].A total of 3853355, 3061761, 5573723, 4534609, 4291129, and 3808590 mappable reads were detected from the intact control, TNBC, PR+BC, TNBC/DCP, PR+BC/CRIZ, and PR+BC/TOP animal samples, respectively. These were mapped to various classes of non-coding RNAs (miRNAs, piRNAs, snoRNAs, snRNAs, rRNAs, and tRNAs) (Table 1). MicroRNAs constituted the largest part of the small RNA pool, reaching 81.7, 77.8, 79.8, 77.9, 72.4, and 82.1% of all small RNA molecules detected in intact controls, TNBC, PR+BC, TNBC/DCP, PR+BC/CRIZ, and PR+BC/TOP samples, respectively (Table 1).

**Table 1:** Library composition

Absolute composition						
CATEGORY	INTACT	TNBC	TNBC/DPC	PR+BC	PR+BC/TOP	PR+BC/CRIZ
number of samples	3	4	4	3	3	3
total	3853355	3061761	5573723	4534609	4291129	3808590
**miRNA**	3148510	2420296	4457646	3537230	3108049	3125959
snoRNA	32438	20672	37850	30798	26334	24089
snRNA	1422	1516	2806	2314	2524	1720
rRNA	21921	16848	29983	22662	21086	18516
tRNA	108118	176533	246617	276325	497035	145168
piRNA	30690	20876	38425	32886	30772	28125
exons	196436	160890	322187	265234	247267	180506
repats	42549	27304	50582	36629	35009	32587
introns	30529	25988	47328	40121	39528	28231
unclassified	22427	15676	30110	24959	22658	19392
no_match	218315	175162	310189	265451	260867	204297

Relative composition						
CATEGORY	INTACT	TNBC	TNBC/DPC	PR+BC	PR+BC/TOP	PR+BC/CRIZ
Total %	100.0	100.0	100.0	100.0	100.0	100.0
**miRNA**	81.7	77.8	79.8	77.9	72.4	82.1
snoRNA	0.8	0.6	0.7	0.7	0.6	0.6
snRNA	0.0	0.1	0.1	0.1	0.1	0.1
rRNA	0.6	0.6	0.5	0.5	0.5	0.5
tRNA	2.8	6.5	4.5	6.2	11.5	3.6
piRNA	0.8	0.7	0.7	0.7	0.7	0.8
exons	5.1	5.5	5.8	5.9	5.8	4.7
repats	1.1	0.9	0.9	0.8	0.8	0.9
introns	0.8	0.9	0.9	0.9	0.9	0.8
unclassified	0.6	0.5	0.5	0.6	0.5	0.5
no_match	5.7	5.8	5.6	5.9	6.1	5.4

### Differential expression of miRNAs in the PFC tissues of tumor-bearing treated and untreated animals

**Figure 1:**
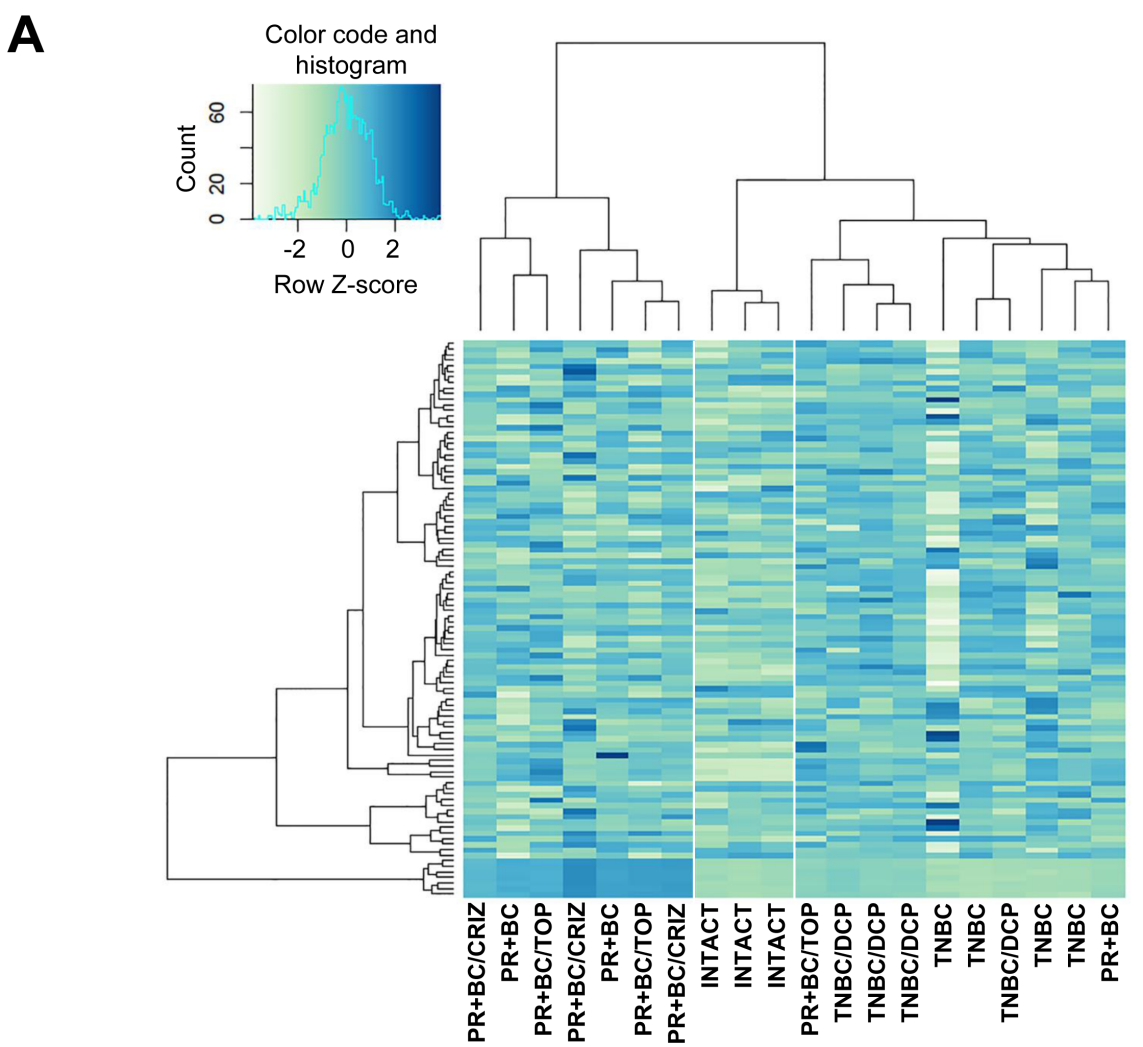

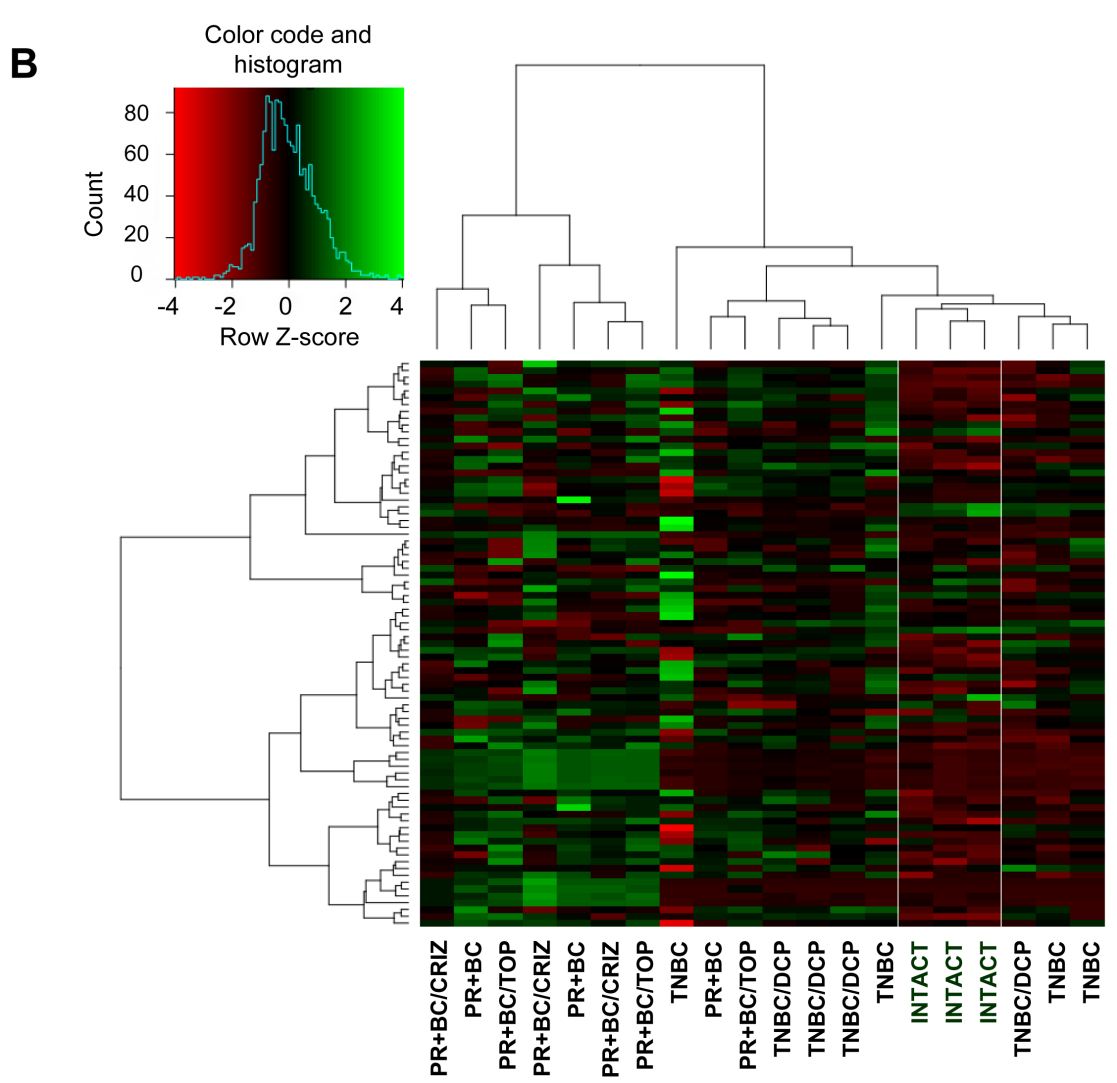
Next generation sequencing-based analysis of small RNA expression in the PFC tissues of intact, TNBC and PR+BC-bearing chemotherapy-treated and untreated TumorGraft mice **A**. Hierarchical clustering of all samples based on the entire small RNA profile. **B**. Hierarchical clustering of all samples based on the microRNAome profile.

We identified all classes of differentially expressed (DE) small RNAs with a fold change > 2.0 and a false discovery rate cut-off of 0.05. Initial unsupervised hierarchical clustering was performed using all DE small RNAs. We noted a distinct separation between intact control PFC tissues and PFC tissues of tumor-bearing animals (Figure 1A, Supplementary Figure 1). Next, we focused on DE miRNA analysis (fold change > 2.0 and false discovery rate cut-off of 0.05) and performed unsupervised hierarchical clustering. Similar to the whole of the small RNAs, we noted a miRNA level-based separation of the intact controls from the samples of tumor-bearing treated and untreated animals, proving that generated miRNA signatures discriminate between sample types (Figure 1B).

While there was an apparent separation between the small RNA and miRNAome profiles of the PFC tissues of the intact control and TNBC- and PR+BC-bearing animals, profiles of the PFC samples of tumor-bearing treated animals differed only from those of intact controls and not from the tumor-bearing untreated animals. Amongst samples, TNBC samples clustered better than the PR+BC samples. These differences may depend upon tumour transplantation, positioning and growth in each individual animal and host responses.

Furthermore, sample clustering revealed they are primarily distinguished by the relative expression of a common set of miRNAs (Figure 1B). To gain further insight into the differentially expressed miRNAs and their potential roles in chemo brain and tumor brain, we proceeded to analyze and compare miRNAs that were differentially expressed in various experimental groups.

**Figure 2:**
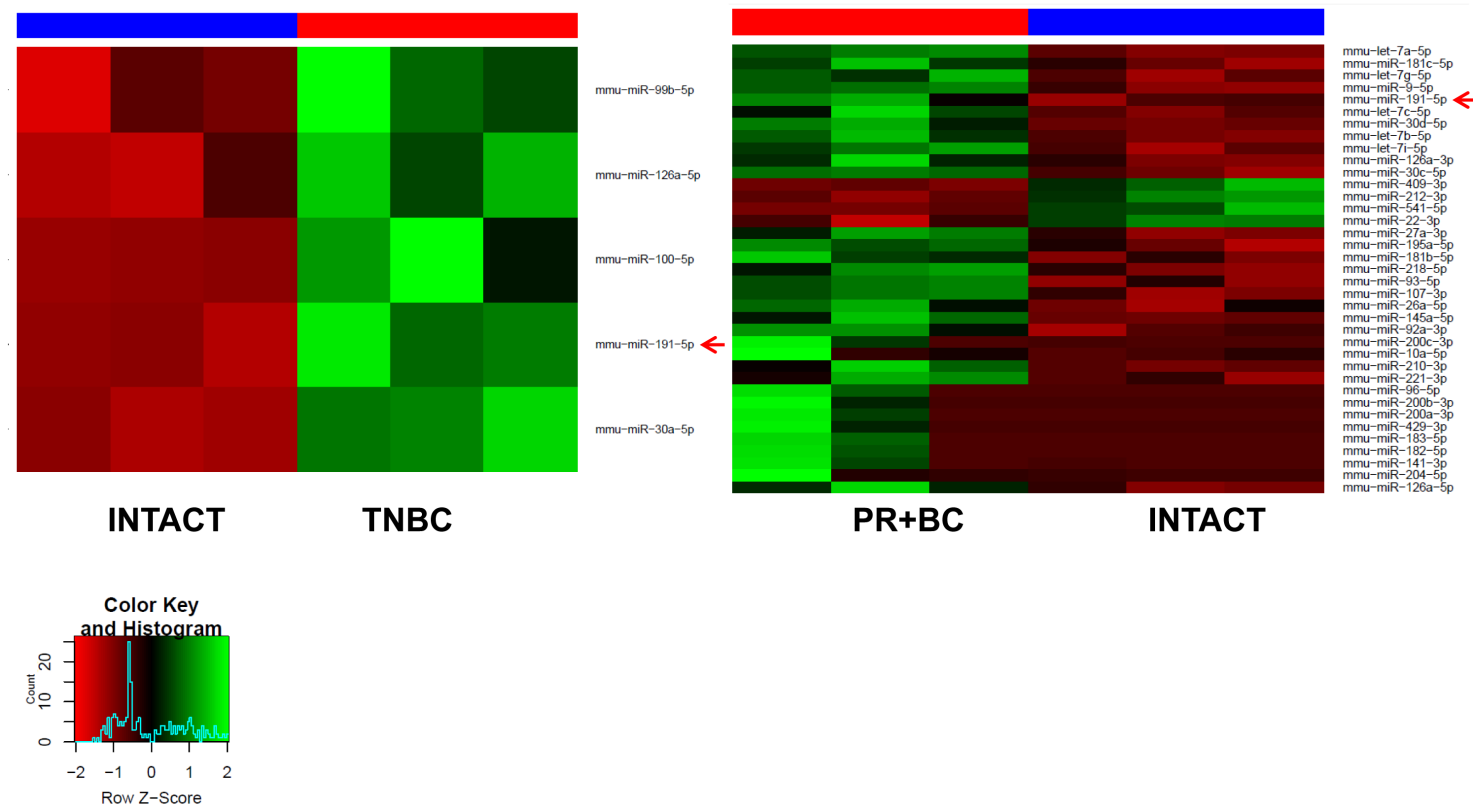
Heatmaps of microRNAs differentially expressed in the PFC tissues of the TNBC and PR+BC tumor bearing animals as compared to intact controls Red arrow indicates miRNAs commonly regulated in both groups.

NGS data revealed that the presence of a malignant extracranial tumor alone had a major effect on the miRNAome. We found that 5 miRNAs were up-regulated in the PFC tissues of TNBC bearing mice, as compared to intact controls. In the PFC tissues of PR+BC bearing mice, 33 miRNAs were up-regulated, and 4 miRNAs were down-regulated. Among those, miR-191-5p was up-regulated in both TNBC and PR+BC tumor-bearing groups (Figure 2).

**Figure 3:**
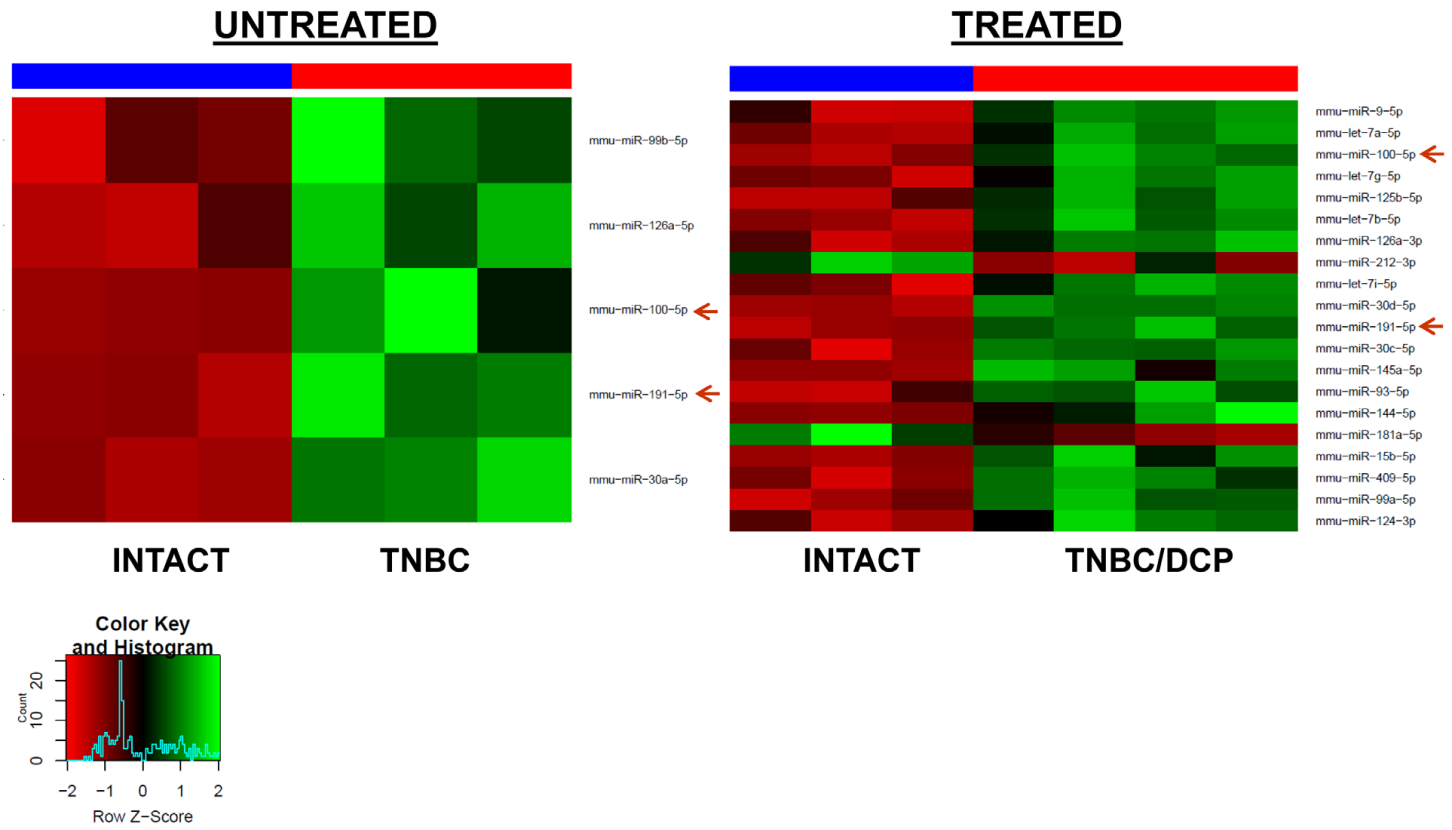
Heatmaps of microRNAs differentially expressed in the PFC tissues of the TNBC untreated and TBNC/DCP mice as compared to intact controls Red arrows indicate miRNAs commonly regulated in both groups.

Treatment of TNBC animals with the DCP regimen led to the upregulation of 18 and downregulation of 2 miRNAs in the PFC tissues of tumor-bearing treated animals. Two miRNAs (miR-191-5p and miR-100-5p) were up-regulated in TNBC and TNBC/DCP animals, as compared to intact controls (Figure 3). Interestingly, no differential miRNA expression was detected between TNBC tumor-bearing treated and untreated animals.

**Figure 4:**
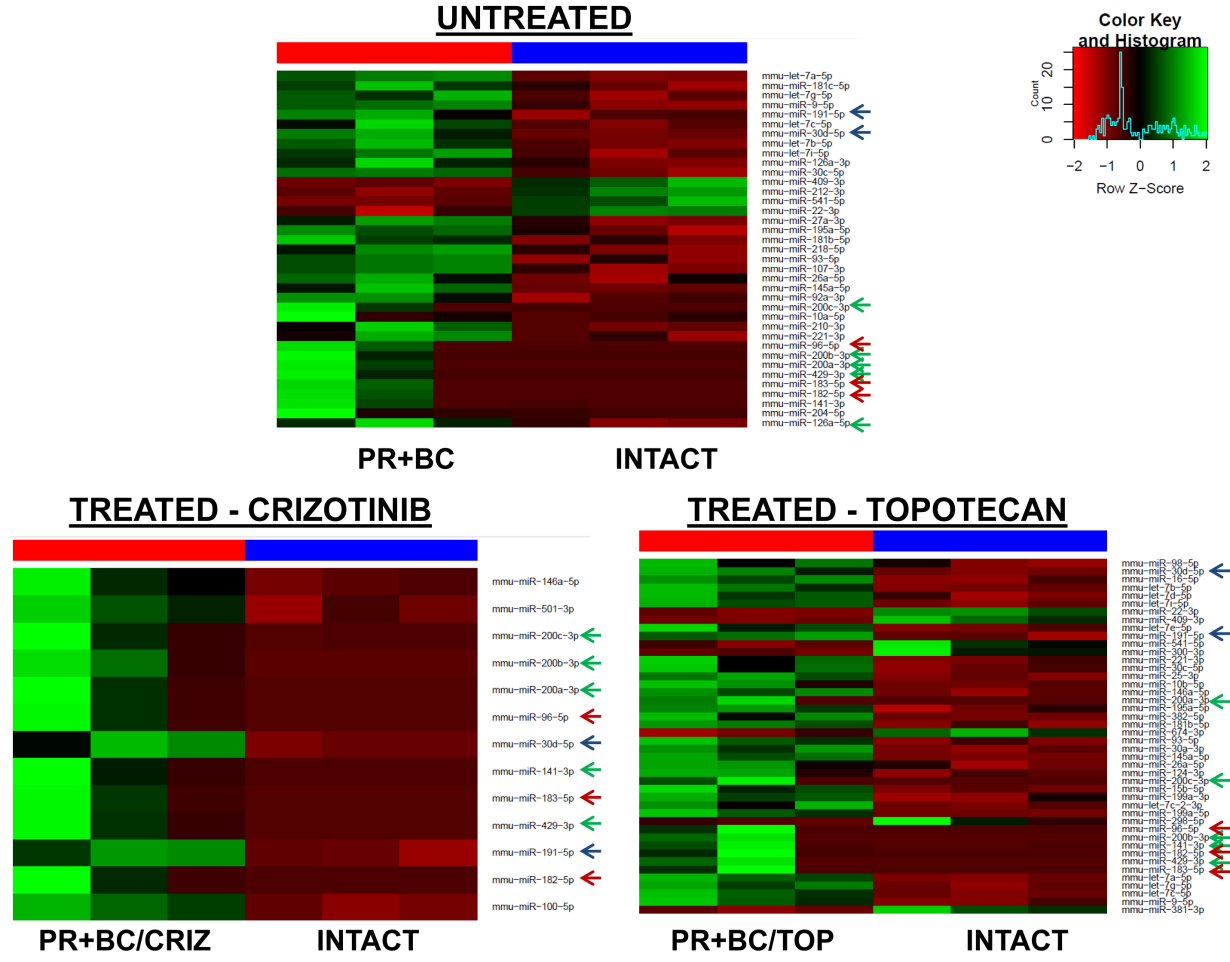
Heatmaps of microRNAs differentially expressed in the PFC tissues of the PR+BC untreated, PR+BC/CRIZ and PR+BC/TOP mice as compared to intact controls Arrows indicate miRNAs commonly regulated in both groups. Green arrow - miR-200 family; blue arrow - miR-183/96/182 cluster; red arrow - other common miRNAs.

The growth of PR+BC and subsequent chemotherapy treatments also affected the miRNAome profiles of PFC tissues of TumorGraft mice. There, treatment of PR+BC animals with crizotinib led to the upregulation of 13 microRNAs in the PFC tissues of experimental animals, as compared to intact controls. Topotecan treatment led to the upregulation of 37 and downregulation of 7 miRNAs (Figure 4).

We noted a commonality between all three groups (PR+BC, PR+BC/CRIZ, and PR+BC/TOP): miR 200 family (miR-200a, miR-200b, miR-200c, miR-141, and miR-429), miR-183/96/182 cluster, miR-30d-5p, and miR-191-5p were up-regulated, as compared to intact controls. Moreover, miRNAs of the let-7 family were up-regulated in the PFC tissues of PR+BC untreated and crizotinib-treated animals as compared to intact controls. MiR-409-3p and miR-22-3p were down-regulated in the PFC tissues of the PR+BC untreated and crizotinib-treated PR+BC animals. Similar to the TNBC TumorGraft mice, no differential miRNA expression was identified between PR+BC tumor-bearing treated and PR+BC tumor-bearing untreated mice.

### Altered levels of BDNF in the PCF tissues of tumor-bearing treated and untreated animals

MiR-191-5p was up-regulated in all animal groups. Interestingly, miR-191 was recently reported to be a regulator of brain-derived neurotrophic factor (BDNF) [51]. BDNF is a member of the nerve growth factor family and one of the fundamental factors regulating neuronal growth, maturation and survival in the brain and spinal cord [52]. Among the other miRNAs, the miR-183/96/182 family and miR-10b target BDNF as well [51, 53]. With this in mind, using western immunoblotting, we analyzed the levels of BDNF in the PFC tissues of intact controls, and TNBC and PR+BC tumor-bearing treated and untreated mice.

**Figure 5:**
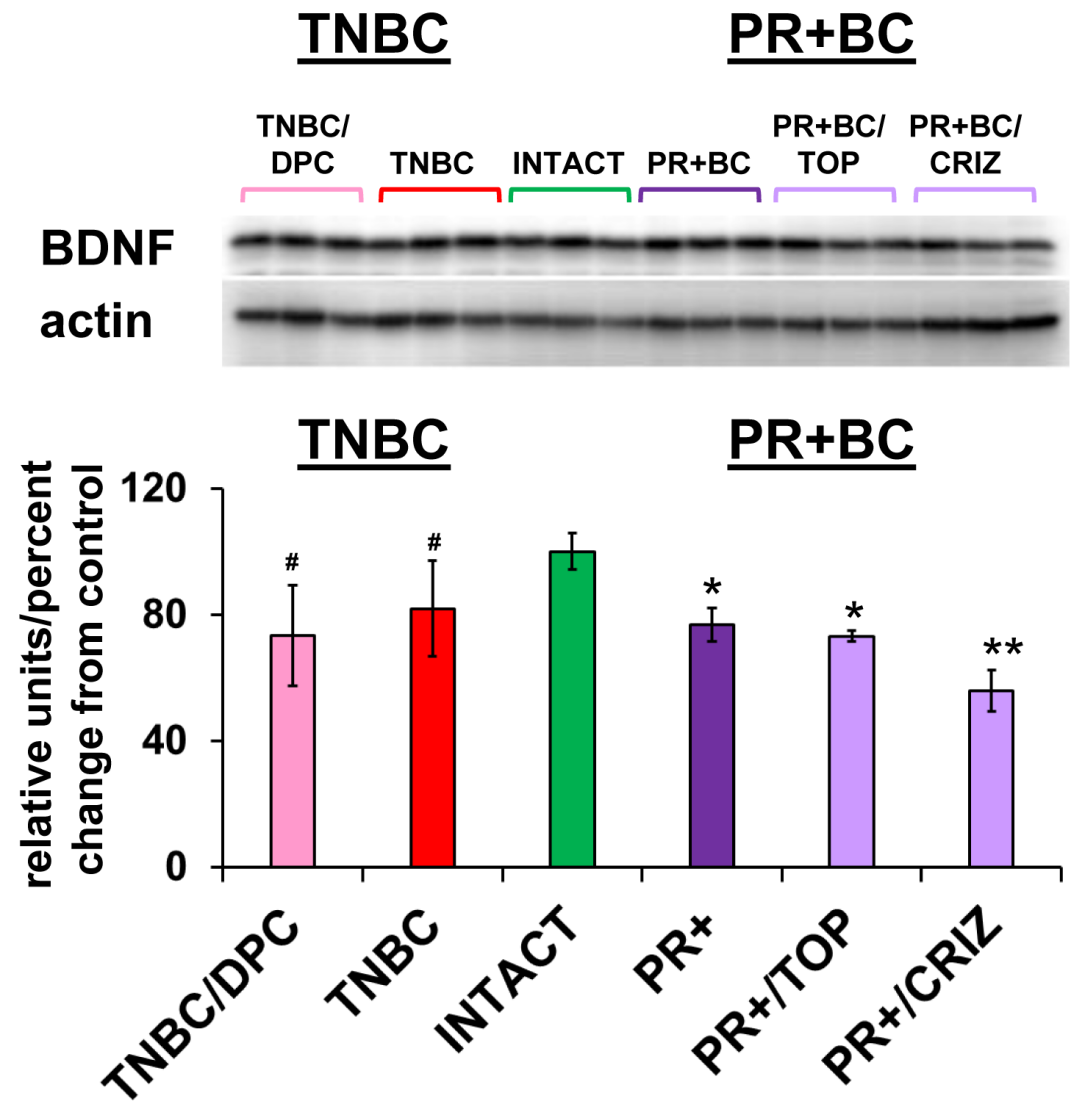
Levels of BDNF in the PFC tissues of intact and TNBC and PR+BC-bearing chemotherapy treated and untreated TumorGraft mice Data are shown as relative units/percent change from control. Due to protein size differences and scarcity of tissue membranes were re-used several times in context of a large scale study. #*p* < 0.10; * *p* < 0.05; ** *p* < 0.01.

BDNF levels were significantly decreased in the PFC tissues of PR+BC TumorGraft animals (*p* = 0.00006) and in PR+BC/TOP and PR+BC/CRIZ (*p* = 0.0192 and *p* = 0.0050, respectively), as compared to intact controls. Moreover, levels of BDNF in the PFC tissues of PR+BC/CRIZ were also significantly decreased, as compared to untreated PR+BC animals (*p* = 0.0011). In the untreated TNBC cohort, BDNF levels exhibited a trend to decrease (90% confidence level). No significant change was observed in the TNBC/DPC animals, albeit a down-regulation trend was noted (*p* < 0.10) (Figure 5).

### MiRNA processing machinery in the PCF tissues of tumor-bearing treated and untreated animals

**Figure 6:**
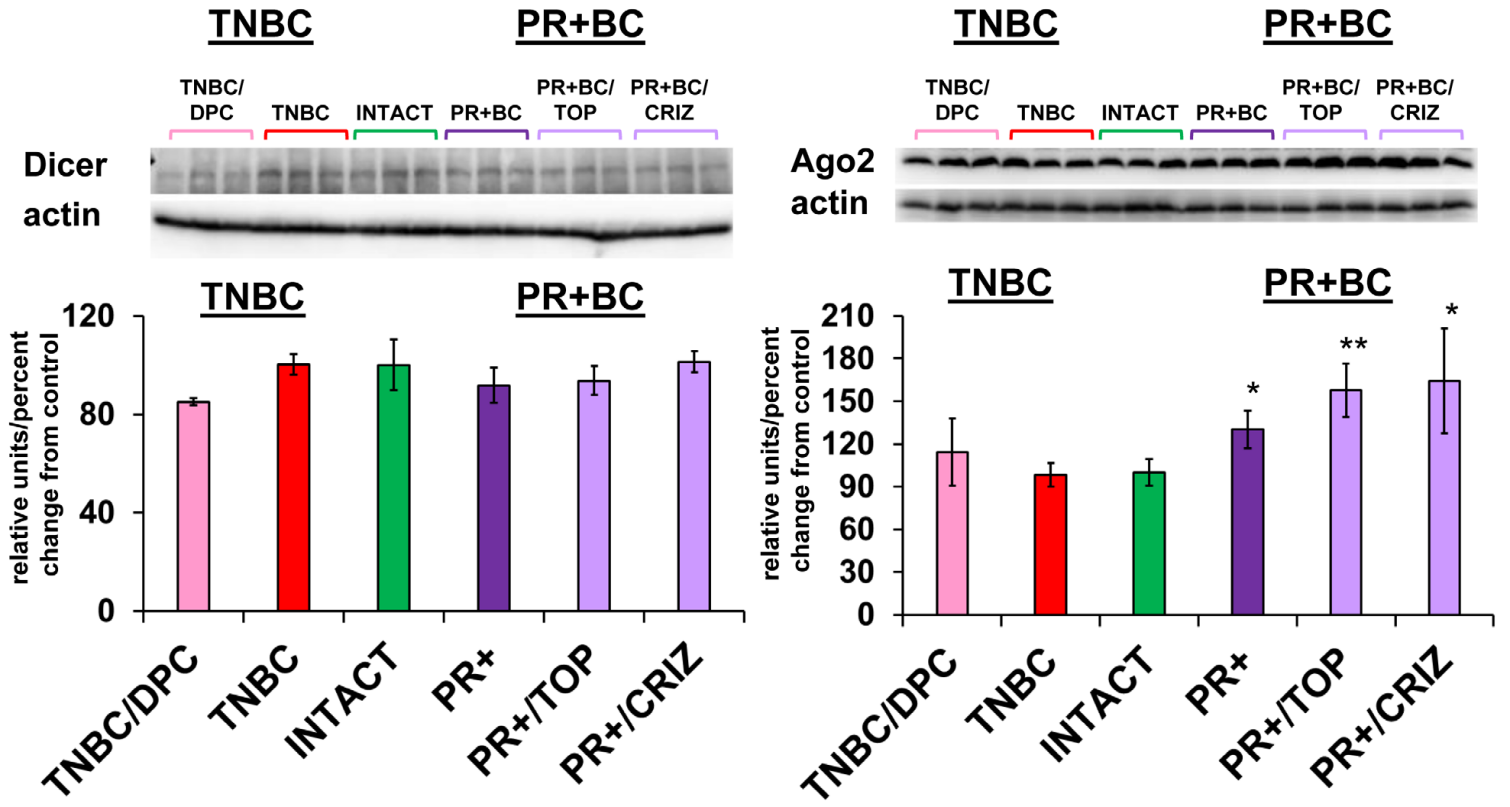
Levels of Dicer and Ago2 in the PFC tissues of intact and TNBC and PR+BC-bearing chemotherapy treated and untreated TumorGraft mice Data are shown as relative units/percent change from control. Due to protein size differences and scarcity of tissue membranes were re-used several times in context of a large scale study. * *p* < 0.05; ** *p* < 0.01.

To determine the possible mechanisms of aberrant miRNAome changes, we analyzed the levels of Dicer and Ago2 proteins - members of small RNA processing machinery. While no statistically significant changes were noted in the levels of Dicer, levels of Ago2 were significantly up-regulated in the PFC tissues of untreated PR+BC TumorGraft animals (*p* = 0.0010) and in PR+BC/TOP and PR+BC/CRIZ (*p* = 0.00005 and *p* = 0.0020, respectively) (Figure 6).

## DISCUSSION

This is the first in-depth analysis of microRNAome profiles in PFC tissues of chemotherapy treated and untreated mouse TumorGraft™ models of triple negative and progesterone receptor positive breast cancer. We saw that the growth of TNBC and PR+BC tumors affects the miRNAome.

While a large body of evidence has accumulated on the incidence of cancer and cancer treatment-related cognitive changes (tumor brain and chemo brain), there is still a lot to learn about their molecular mechanisms. As per the timing and long-term persistence of tumor brain and chemo brain, these phenomena might be epigenetic in nature and based on altered gene expression patterns affecting brain function.

Epigenetic changes governing alterations in gene expression include DNA methylation, histone modification, and small RNA-associated silencing. Amongst the latter, miRNAs (evolutionarily conserved, small, single-stranded RNA molecules that operate as main negative gene regulators) are of special interest and significance. MiRNAs are important in brain development and in all CNS functions and processes. They regulate cellular proliferation, differentiation, repair and death, and take part in inflammatory responses. Aberrant expression of miRNAs underlies a wide array of neurological and psychiatric diseases, such as Alzheimer’s, Parkinson’s, and Huntington’s, stroke, traumatic brain injury, amyotrophic lateral sclerosis, autism, schizophrenia, and brain tumors [54].

Even so, nothing is known about the role of miRNAs in the brain’s response to the combination of malignant non-CNS tumor growth and subsequent chemotherapy. This is the first study to analyze miRNA changes in tumor brain and chemo brain. We showed that TNBC and PR+BC tumor growth and presence significantly altered the miRNAome in the PFC tissues of TumorGraft animals. The PFC is a key regulatory region that receives input from all other cortical areas, and serves to coordinate executive function, motor, cognitive, and social behaviors, attention, and working memory [55, 56].

While tumor growth-related miRNAome changes were observed in the PFC tissues of both breast cancer groups, they were more pronounced in the PFC tissues of PR+BC-bearing animals than in TNBC-bearing ones. The reasons behind these changes and their molecular mechanisms, along with potential cellular changes and behavioral repercussions need to be further studied and analyzed, and may depend upon the biology of tumors as well as chemotherapy responsiveness. Chemotherapy treatment of TNBC and PR+BC-bearing animals led to further alterations in the miRNAome, and amongst differentially expressed miRNAs, miR-191-5p was up-regulated in all experimental groups (tumor-bearing treated and untreated groups, as compared to intact controls). While this miRNA has not been extensively researched, some studies have reported that miR-191-5p was up-regulated in major depressive disorders and down-regulated in Alzheimer’s disease (AD) [57]. This miRNA is commonly down-regulated in the blood and plasma of AD patients and is considered to be a circulating AD biomarker [58]. Overall, miRNA expression patterns in the blood and brain do not always fully correlate. Therefore, it would be interesting to analyse the levels of miR-191-5p in the plasma and whole blood of tumor brain and chemo brain animals. This would help to better discern miR-191-5p’s roles in tumor brain and chemo brain as a potential molecular driver and biomarker of the two phenomena. Likewise, it would help determine if there exists any long-distance signalling between the tumor and the brain that involves miR-191-5p or any other mRNAs. New studies reported the role of miR-191-5p in systemic inflammatory response syndrome [59]. Based on that, a potential next step is analyzing the links, if any, between miR-191-5p expression and inflammation in chemo brain and tumor brain - especially in light of evidence revealing the role of inflammation in chemo brain [60].

Along with miR-191-5p, miR-100-5p was upregulated in TNBC animals and TNBC/DCP animals, as compared to intact controls. Recently, miR-100-5p was shown to be an upregulated circulating marker of Huntington’s disease [61]. Furthermore, this miRNA was said to be involved in the amyloid β-induced pathologes [62], whereby, along with miR-99b-5p, it affected neuronal survival by targeting PI3K/Akt/mTOR. In Alzheimer’s disease (AD), miR-100-5p was downregulated in the early-middle stages,, but upregulated at the late stages of the disease. Activation of PI3K/Akt signaling promotes neuron survival. Downregulation of miR-100-5p at the early stages of AD may protect neurons from amyloid β-induced apoptosis, while such protection can be lost at later stages when miR-100-5p gets upregulated [63]. More studies are needed to dissect the roles of miR-100-5p and PI3K/Akt/mTOR in tumor brain and chemo brain and how they relate to the changes induced by TNBC tumor growth and DCP treatment.

DCP treatment caused upregulation of the let-7 family miRNAs - let-7a, 7b, 7g, 7i - in the PFC tissues of TBNC mice, as compared to intact controls. These family members were also upregulated in the PFC tissues of PR+BC-untreated (let-7a, 7b, 7c, 7g, 7i) and PR+BC/topotecan-treated mice (let 7a, 7b, 7c, 7d, 7e, 7g, 7i). The let-7 family is highly conserved in both invertebrates and vertebrates, and was the second such miRNA family to be identified and characterized [64]. Since the different let-7 family members have similar or even identical seed sequences, they likely have overlapping sets of target mRNAs. The let-7 family is one of the well-known tumor-suppressor miRNA families [65, 66] that target RAS [67, 68], a cellular oncogene. Many tumors exhibit profound downregulation of let-7 [69], and the overexpression of let-7 strongly suppresses tumor cell growth. Let-7 levels are usually low in undifferentiated cells. In lung cancer, low let-7 levels are correlated with poor survival [70].

The let-7 family is involved in neural development and neuronal differentiation [24, 71]. In the brain, let-7 levels have been shown to increase after cerebral ischemia/reperfusion injury [72, 73], whereas let-7 suppression inhibited apoptosis and inflammatory responses and caused an overall neuroprotective effect upon cerebral ischemia/reperfusion injury [73]. In a rat model of a middle cerebral artery occlusion and subsequent reperfusion injury, let-7 family members were upregulated at the 48-hour reperfusion time point [72, 74]. In *C. elegancs,* several neurons have shown increased levels of *let-7* as they age, whereby let-7 upregulation contributed to the decline of aging neurons’ regeneration potential [75]. Let-7 microRNAs was downregulated in radiation-exposed neural granule cell progenitors, as well as in medulloblastoma [76]. Several other studies have shown radiation-induced downregulation of let-7 family members in cells and tissues, and this downregulation was associated with the altered expression of the DNA damage mediator protein p53 [77, 78]. The let-7 family is also involved in glioma, where the upregulation of let-7b inhibited proliferation, migration and invasion in glioma cell lines. Furthermore, increased levels of let-7b reduced the stemness of glioma stem-like cells [79]. Overall, upregulation of the let-7 family may viewed as a positive and protective event in the context of cancer and brain metastasis, whereby it acts as a tumor-suppressor and blocks proliferation. Contrarily, in the context of some brain diseases and damage, it may have negative effects as well, as glial proliferation is important in repairing tissue damage. In the future, more studies are needed to dissect the cellular, tissue-specific, and behavioral repercussions of let-7 upregulation in tumor brain and chemo brain.

The most pronounced miRNAome changes were observed in the PR+BC group. PR+BC tumor growth and subsequent chemotherapy treatment with topotecan and crizotinib had profound effects on the miRNAome of TumorGraft animals’ PFC tissue. Both tumor growth and chemotherapy treatment led to the upregulation of the miR-200 family, as compared to intact animals. We observed the upregulation of eight miRNAs that make up a related miR-200 family (miR-200a, 200b, 200c, miR-141, miR-429) and a miR-183/96/182 cluster. These miRNAs are often co-transcribed and referred to as the miR-200/182 cluster. It was shown the entire miR-200/182 cluster is upregulated in acute herpes simplex virus 1 encephalitis [80].

The miR-200 family is important for the proper balance between neuronal proliferation and differentiation during development [81]. Its aberrant expression has been associated with various types of malignant tumors [82], and was correlated with drug resistance and patients’ overall survival [83]. A recent study analyzed and compared the miRNA profiles of gastric adenocarcinomas and brain metastatic carcinomas and identified the upregulation of, among others, the miR-200 family members miR-141-3p and miR-200b-3p in the brain metastatic samples [84]. Therefore, deregulation of the miR-200 family could be involved in brain metastasis *via* the Zeb/miR-200 family feedback loop. Overall, much remains to be discovered about the miR-200 family’s roles in various diseases and conditions, including cancer and cancer treatment-associated CNS toxicity. The miR-183/96/182 cluster upregulated in the PR+BC tumor-bearing treated and untreated mice was implicated in hepatocellular carcinoma, breast cancer, and glioma [85-87]. It has also been linked with light-induced retinal injury and was shown to target brain-derived neurotrophic factor (BDNF) [53]. BDNF is also a target of miR-191 that was upregulated in all experimental animal groups, as compared to controls [51].

The negative correlation between the levels of miRNAs that target BDNF and the levels of BDNF protein constitutes an interesting and important finding. We noted that BDNF levels were strongly downregulated in PR+BC tumor-bearing treated and untreated animals; however, TNBC treated and untreated animals exhibited only a trend. BDNF, along with the nerve growth factors neurotrophin-3 and neurotrophin-4/5, is a member of the neurotrophin family [88]. BDNF is a key regulator of neural development, survival, growth, differentiation and plasticity [89, 90] because of its involvement in controlling the expression of pro-survival and anti-apoptosis genes [88]. Furthermore, BDNF is known to modulate synaptic function and plasticity, and is involved in learning and memory [88]. Deletion of the BDNF gene causes dendritic degeneration and neuronal loss, and decreased BDNF levels are associated with cognitive impairments in patients with Parkinson’s disease [91], Alzheimer’s disease [92], depression, and many other neurological and psychiatric disorders. A missense mutation in BDNF was shown to alter cognitive performance post-traumatic brain injury [93]. As one of the main neurotrophic factors, BDNF constitutes a promising remedy for reducing neuronal injury after cerebral ischemia [94] and for functional recovery in ALS [95].

It was reported that depression, which might be a manifestation of tumor brain and chemo brain in cancer patients, is often associated with reduced BDNF serum levels [96, 97]. Low levels of BDNF in cancer patients were connected with depression and poor prognosis^96^. However, an animal model-based study of doxorubicin and cyclophosphamide chemotherapy-induced chemo brain showed that behavioral deficits (anxiety and spatial cognition impairments) were paralleled by decreased neurogenesis and lowered serum BDNF levels without alterations to BDNF, mRNA, or protein levels in the brain [98]. In our study, we saw significant downregulation of BDNF in PR+BC tumor-bearing treated and untreated mice and a trend towards downregulation in tumor-bearing TNBC animals. Hence, BDNF downregulation may be due to the presence of non-CNS malignant tumors, rather than chemotherapy treatment itself. In context of previous studies, BDNF down-regulation may have negative consequences for both brain and behaviour. Going forward, we would seek to examine the mechanisms of tumor and chemotherapy-induced effects on BDNF levels in conjunction with miRNA. And, in addition, analyze the mechanisms regulating miRNA expression in the brains of tumor-bearing treated and untreated animals, as well as the neuroanatomical and behavioral repercussions of tumor and chemo brain-associated BDNF downregulation. Furthermore, it would be important to establish and compare the roles of tumor growth and chemotherapy in BDNF regulation, and analyze BDNF regulation as a function of malignant tumor load. As such, successful chemotherapy leads to a reduced tumor load, which by itself may affect BDNF levels.

While we did not see any changes in the Dicer levels, we noted a significant upregulation in the levels of Ago2 in the PFC tissues of PR+BC tumor-bearing treated and untreated animals. Elevated Ago2 levels may, in turn, contribute to the more profound changes in miRNA expression observed in the PR+BC groups. Upregulation of the Ago2, protein that partakes miRNA production and execution of miRNA-mediated gene silencing was implicated in the regulation of cocaine addiction and anorexia [99]. Moreover, increased levels of Ago2 conveyed very poor prognosis in glioma [100]. The mechanisms of Ago2 upregulation and its repercussions in tumor brain and chemo brain need to be further studied.

Here we focused on tumor brain and chemo brain as induced by the growth of TNBC and PR+BC tumors, both stage IV. In the future, it would be prudent to look at the miRNAome as a function of tumor stage and grade, as well as to analyse miRNAome deregulation caused by other tumor types. Additionally, we focused our attentions on the PFC; however, previous studies have suggested chemo brain also manifests in the hippocampus [98]. Therefore, it would be interesting to analyze the brain region specificity of chemo brain and tumor brain and to correlate those findings with the roles of miRNAs.

**Figure 7:**
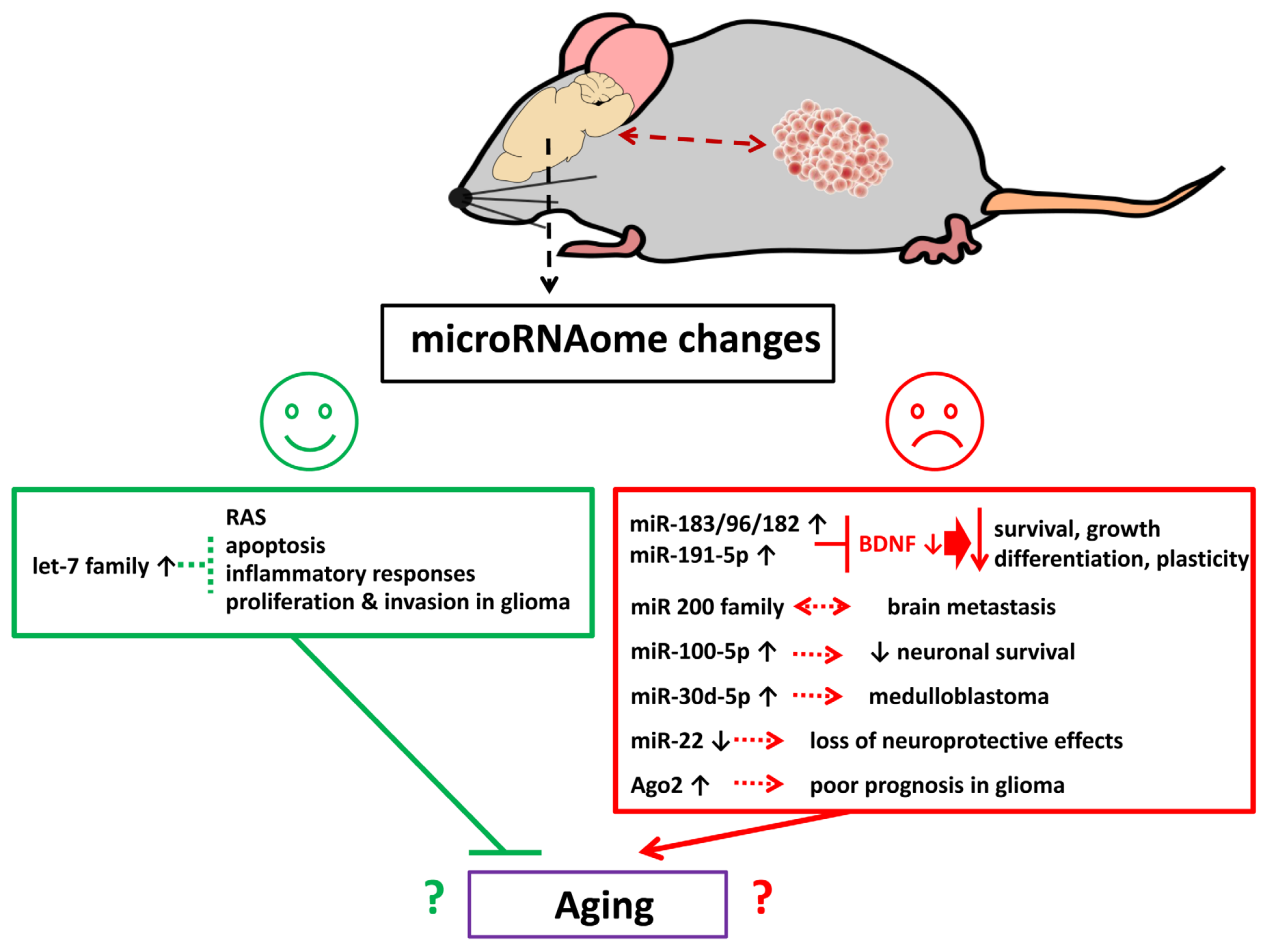
Schematic representation of possible biological effects of tumor brain and chemo brain-induced miRNAome changes in the brain

In sum, growth of malignant TNBC and PR+BC tumors altered the miRNAome of tumor bearing treated and untreated animals’ PFC. The observed changes may have opposing functional consequences - some positive and protective and some, negative and deleterious. Among altered miRNAs, miR-183/96/182 cluster and miR-191-5p both target and downregulate BDNF. Here their increased expression was paralleled by a decrease in BDNF. Low BDNF levels have been shown to decrease neuronal survival, growth, differentiation and plasticity, and were reported in a wide array of neurological diseases and conditions. Meanwhile, miR-22 is a well-studied neuroprotective molecule [101] and its observed downregulation may have detrimental consequences for cells. The miR-200 family was associated with brain metastases [84], and miR-30d-5p was implicated in medulloblastoma development [102]. Furthermore, Ago2 protein upregulation is a very negative glioma prognostic factor [100] (Figure 7).

Some of the observed miRNA changes could be associated with cellular senescence and aging. Amongst these, oxidative stress-induced upregulation of the miR-200 family caused senescence [103]., and stress-induced cellular senescence was also promoted by the miR-183/96/182 cluster [104]. MiR-191-5p is one of the biomarkers of aging-associated Alzheimer’s disease. These miRNAs therefore ought to be explored as potential drivers of aging processes. On the other hand, the let-7 family includes many potent senescence-inhibiting and anti-age miRNAs [105]. Overall, there may be complex interplay between miRNA-mediated pro-and anti-senescence pathways and processes in the PFC tissues of tumor-bearing animals. The precise nature, regulation, and organismal and behavioral repercussions of altered expression in these miRNA families and clusters needs to be looked (Figure 7).

For a bigger picture of tumor brain and chemo brain’s mechanisms, we would proceed with identifying molecular pathways that were aberrantly expressed and regulated by miRNAs As such, it would be prudent to analyse the mechanisms, timing, and potential feedback loops between miRNA and mRNA regulation and expression, and integrate transcriptome, small RNAome, as well as methylome. More studies are needed to dissect the effects of miRNAs on signalling pathways and to discern the interplay between the various signalling pathways underlying tumor brain and chemo brain in the context of the entire signalome and interactome, as well as their functional outcomes. We focused on miRNAs, molecules that constitute the largest proportion of differentially regulated small RNAs. In the future, we would seek to analyze the roles of other small RNA molecules, such as tRNAs, snoRNAs, snRNA and others. These could prove to be important tumor brain and chemo brain biomarkers. In summary, this study is the first to show miRNAome deregulation in tumor brain and chemo brain, and may serve as a roadmap for further analysis of cancer and chemotherapy’s neural side effects. In the future, in order to gain a full understanding of tumor brain and chemo brain, molecular and cellular data need to be put in context of neuronal structure analysis and behavioral changes.

## MATERIALS AND METHODS

### Animal model

We used the well-established TumorGraft technology developed by Champions Oncology, Inc. (Baltimore, MD); the frozen brain tissues of TumorGraft mice carrying TNBC and PR+BC patient-derived xenografts (PDX) were provided by Champions Oncology, Inc. Patients diagnosed with TNBC and PR+BC had their tumors engrafted into mice to generate personalized TumorGraft mouse models for precision oncology approaches. The patients provided their informed consent, which covered the use of tumor material for research purposes. TumorGrafts were generated, as previously described [106-110]. In brief, a fresh specimen of the tumor is obtained during surgery, and small fragments of the tumor containing malignant cells and supportive stromal components are inserted subcutaneously into the flanks of six-week-old immunodeficient female mice (female *nu*/*nu* athymic mice; Harlan Laboratories, Indianapolis, IND) and further propagated as earlier described. Animal experiments were approved by Institutional Animal Care and Use Committee protocols. Following tumor propagation, when TumorGrafts grew larger than 200 mm^3^, the animals were divided into groups of three to five. Drug dosage and drug combinations were applied consistent with individual physicians’ recommendations and in consultation with the patient. The starting volumes differed between individual TumorGraft models because of the varying doubling time involved. As per agreed protocol, TNBC PDX-bearing TumorGraft animals were treated with Doxorubicin/Paclitaxel/Cyclophosphamide (*n* = 4 treated and 4 untreated), and PR+BC animals with Topotecan (PR+BC/TOP) or Crizotinib (PR+BC/CRIZ) (*n* = 3 treated and untreated). Intact animals (no tumour, no treatment, *n* = 3) served as baseline controls. The aforementioned chemotherapy agents were prepared according to the manufacturer’s specifications, and Champions Oncology ran all chemotherapy treatments. Tumor growth was strictly monitored; tumor dimensions were regularly measured and tumor volumes were calculated as previously described [110]. In both cases, chemotherapy applications resulted in successful tumor suppression and reduction of tumor growth (data not shown). Animals were humanely sacrificed; the brains were removed and immediately frozen in liquid nitrogen and stored in −80 °C for molecular analysis. The tissues were split to accommodate DNA, RNA, and protein analysis.

### Total RNA isolation and small RNA sequencing

Small RNA sequencing was conducted using Illumina next generation sequencing technology as previously described [111]. In brief, total RNA from the PFC tissues was isolated using the RecoverAll Total Nucleic Acid Isolation Kit (Life Technologies). RNA quality and quantity were analyzed with Bioanalyzer 2100 and RNA Nano Chips (Agilent Technologies). Small RNAs were sequenced using TruSeq Small RNA Sequencing Kit (Illumina), TruSeq SR Cluster Kit v5-CS-GA (Illumina) and TruSeq SBS Kit v5-GA (Illumina) according to manufacturer’s instructions. All the samples were sequenced on the Illumina Next 500 sequencer using the 36-cycle single-end protocol. Base calling and demultiplexing were done using CASAVA 1.8.2 with default settings, followed by trimming of adapters using Cutadapt version 1.8.dev0 ( https://cutadapt.readthedocs.io/en/stable/ ). The quality of the sequenced reads after adapter trimming was assessed using FASTQC software (http://www.bioinformatics.babraham.ac.uk/projects/fastqc/). The bowtie v.1.1.2 version [112] was used to map the reads to the reference mouse genome (UCSC mm10 genome assembly). Trimmed reads were sequentially mapped to various small classes (miRNAs, snRNA, rRNA, snoRNA, tRNAs) and genomic features (repeats, exons and introns), reads that did not map to any of small RNAs or other genomic features were considered unclassified. Further analysis focused on miRNA group. To detect differentially expressed small RNAs. Raw counts of unique tags were loaded in R. Normalization and the detection of differentilly expressed tags was done using DESeq2 Bioconductor package [113]. Multiple comparisons adjustment was done with Benjamini-Hochberg procedure.

### Western immunoblotting

Western immunoblotting was conducted as previously described [114-117]. In brief, around 50 mg of PFC tissues were sonicated in ice-cold 1% SDS and immediately boiled. Protein concentrations were determined using the Bradford assay (BioRad, Hercules, CA). Equal amounts of protein (10-30 μg) were separated by SDS-PAGE into slab gels of 10-15% polyacrylamide and transferred to polyvinylidene difluoride membranes (Amersham Biosciences, Baie d’Urfé, Quebec). Eight membranes were prepared in total. Due to scarce amount of tissues, membranes were re-used and re-probed to allow for analysis of miRNA machinery and targets (this study), as well as epigenetic regulators (Kovalchuk et al., 2017, Aging, in press). The membranes were incubated with primary antibodies against BDNF, Dicer and Ago2 (1:1000, Abcam), and actin (1:2000, Abcam) overnight at 4° C. Primary antibody binding was detected using horseradish peroxidase-conjugated secondary antibodies and the Enhanced Chemiluminescence Plus System (Amersham Biosciences, Baie d’Urfé, Quebec). Chemiluminescence was detected using a FluorChem HD2 camera with FluorChem software (Cell Biosciences). The membranes were stained with Coomassie blue (BioRad, Hercules, CA) to confirm equal protein loading. Signals were quantified using NIH Image J64 software and normalised relative to actin or Coomassie staining.

### Statistical analyses

Statistical analysis (Student’s t-test) was performed using the Microsoft Excel software package.

## SUPPLEMENTARY MATERIALS FIGURE


