# Supplementary material for: Growth of malignant extracranial tumors alters microRNAome in the prefrontal cortex of TumorGraft mice
Source: Oncotarget. 2017 Aug 3;8(51):88276–93. doi: 10.18632/oncotarget.19835 (PMC5687604; doi:10.18632/oncotarget.19835)
Supplement: Supplementary file 1 [file oncotarget-08-88276-s001.pdf]

Growth of malignant extracranial tumors alters microRNAome in the prefrontal cortex of TumorGraft mice

SUPPLEMENTARY MATERIALS

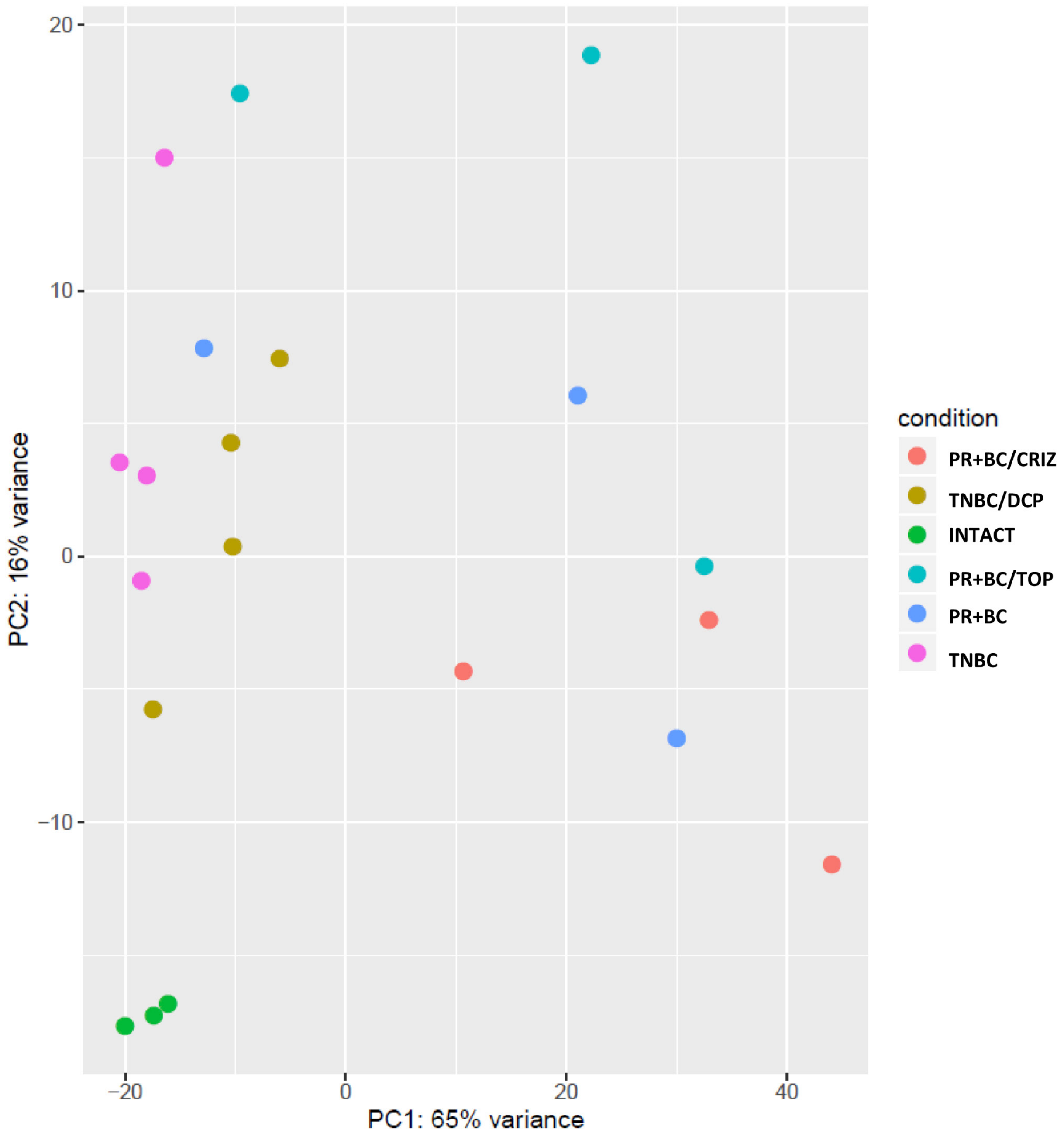

Supplementary Figure 1: MDS plot based on the expression of all of the detectable small RNAs.
